# Fluorescence polarization modulation super-resolution imaging provides refined dynamics orientation processes in biological samples

**DOI:** 10.1038/s41377-022-01018-w

**Published:** 2022-11-07

**Authors:** Sophie Brasselet

**Affiliations:** Aix Marseille Univ, CNRS, Centrale Marseille, Institut Fresnel, F-13013 Marseille, France

**Keywords:** Polarization microscopy, Super-resolution microscopy

## Abstract

Combining polarization modulation Fourier analysis and spatial information in a joint reconstruction algorithm for polarization-resolved fluorescence imaging provides not only a gain in spatial resolution but also a sensitive readout of anisotropy in cell samples.

## Abstract (web summary)

Polarization-modulated fluorescence microscopy provides molecular orientation mapping in cells.

## Graphical abstract

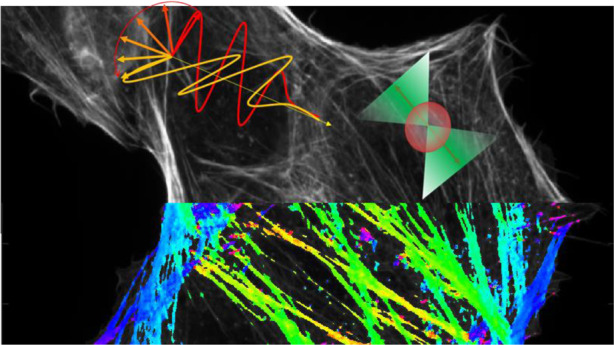

Cells and tissues are made of complex assemblies of proteins and lipids, which nanoscale structure is intrinsically related to their functions. Studying such structures is determining to understand pathologies, however, they cannot be accessible via standard fluorescence microscopy imaging due to their sub-diffraction size. Despite the great progresses made in super-resolution fluorescence imaging to surpass the diffraction scale (~200 nm), an important missing information to reveal such complex architectures is the orientation of proteins and lipids. Orientation is accessible via the readout of fluorescence sensitivity to polarization, typically through the measurement of a signal modulation upon the rotation of a linear excitation polarization. Fluorescence Polarized Microscopy (FPM) has permitted to quantify the degree of anisotropy and orientation of fluorescent labels in lipid membranes^1^, cytoskeleton proteins in yeast^2^ and cells^3^, membrane proteins in nuclear pore complexes^4^ and integrin^5^, and aggregates such as amyloids^6^. Achieving high polarization sensitivity, however, requires labelling protein domains in a rigid way to limit the orientational fluctuations of the fluorescent label^3,5^, which is not always accessible. FPM is also limited to the diffraction scale, which does not permit the study of fine structure organizations, typically occuring in cell organelles made of folded or highly curved membranes.

Exploiting light polarization to additionally improve spatial resolution in fluorescence imaging is a topic of increasing interest. The fluorescence modulation provided by a rotating linear polarized excitation is indeed an additional parameter that, despite spatial convolution, provides a finer spatial signature of molecular organization, which is sensitive to sub-diffraction scales. To achieve super resolution in polarization microscopy, super-resolution imaging through sparse deconvolution of polarization-modulated fluorescent images (SPoD) has been first implemented, based on a sparsity penalty-enhanced estimation by demodulation (SPEED) algorithm^7^. This method however does not give access to molecular orientation, and raised questions as to whether the nature of the resolution gain is due to polarization modulation. Later a method called super-resolution dipole orientation mapping (SDOM)^8^ demonstrated that the combined implementation of spatial deconvolution and polarization modulation analysis can provide orientation and intensity information beyond the diffraction limit. SDOM still uses, similarly as SPoD, a sparsity constraint hypothesis of the sample and the need to tune regularization parameters, however, it provides quantitative information of the signal modulation. In parallel with these developments, other demonstrations of super-resolution orientation imaging have been developed, including Polarized Structured Illumination Microscopy (pSIM)^9^, Single Molecule Orientation and Localization Microscopy (SMOLM)^10–13^, or the use of other optical contrasts such as Raman imaging^14^ or two-photon nonlinear coherent imaging^15^.

In the article in *Light: Science & Applications*^16^, M. Guan et al. implement a novel analysis approach, termed optical lock-in detection super-resolution dipole orientation mapping (OLID-SDOM), to push forward the capabilities of dynamic, super-resolved orientation imaging in pure wide field FPM. The algorithm is based on a joint reconstruction of both spatial and polarization modulation information without involving sparsity constraints. The image reconstruction relies on an optimization problem that discriminates the modulated and the unmodulated contributions of the signal. The speed of the algorithm is improved by using Fast Fourier Transform phase extraction of the modulation, providing additionally similar denoising advantages as in a lock-in process and fast data processing as compared to iterative algorithms, such as proposed in a related approach^17^. Ultimately, both phase and amplitude of the polarized fluorescence modulation are retrieved, providing both molecular orientation and degree of orientational order (named Orientation Uniform Factor) in a super-resolved image. The technique can achieve 100 frames per second, and resolution improvement with orientations distances down to 50 nm scales, with possible extension to 3D imaging.

**Figure:**
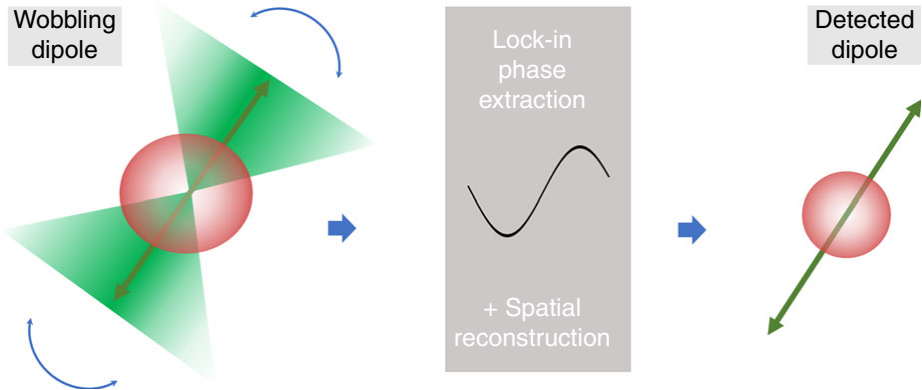
The schematic principle of OLID-SDOM

The striking advantage of the approach is a gain in background rejection, with reduced artifacts under low signal-to-noise ratio (SNR) and therefore ability to measure orientation information under extremely low anisotropy conditions. This appears to be particularly relevant in situations where the polarization modulation is very weak, where the image is not sparse (e.g. as in dense biological samples), or if the image is polluted by important background signal, which is often the case in wide-field microscopy. The most remarkable result found is that even in GFP labelled samples that a priori do not exhibit a particular strategy to minimize the wobbling of the label fluorescent protein, a weak fluorescence anisotropy is found. This finding makes orientation mapping a promising and interesting tool to not only understand the fundamental processes at the origin of this anisotropy, but also image biological processes in a large number of subcellular structures with high spatial resolution.

The authors imaged different subcellular organelles in yeast encoded with specific GFP-tagged proteins; in lipid particles and vesicles, the Golgi complex, actin, spindle pole and endosome. They also demonstrated high-resolution imaging of mitotic spindles and microtubules, as well as a two-color image of F-actin bundle structures together with weakly anisotropic lysosomes. At last, the ability of OLID-SDOM to produce super-resolution images with a high temporal resolution permitted the imaging of highly dynamic cellular processes such as nanoscale morphological changes of mitochondria, which is an important readout of energy regulation in cells. Millisecond-scale remodeling and growth of sub-mitochondrial regions could be in particular reported, as well as time-lapse orientation dynamics of dendritic spines in live neurons in interconnected networks.

OLID-SDOM is thus a promising tool to study highly resolved spatio-temporal structural information in cells, in particular in neuron where synaptic activity is dynamic. With the recent efforts made in developing strategies to minimize orientational fluctuations of genetically encoded fluorescent protein labelling^3,5^, this makes the potential of this method even stronger due to its sensitivity to small anisotropy changes. Other methods could benefit from the data processing presented in this approach, such as pSIM which intrinsically uses super-resolution imaging^9^, sectioned spinning disk confocal polarized microscopy^18^, or fast polarized nonlinear scanning microscopy^19,20^. At last, the achievements shown in this work demonstrate the high potential and promising future of polarized microscopy for biological imaging, even though challenges remain open such as the monitoring of 3D orientations in dense samples, a topic so far only addressed at the single molecule scale^10–13^.
